# Contribution of three-dimensional conformal intensity-modulated radiation therapy for women affected by bulky stage II supradiaphragmatic Hodgkin disease

**DOI:** 10.1186/1748-717X-8-112

**Published:** 2013-05-02

**Authors:** Delphine Antoni, Shanti Natarajan-Ame, Philippe Meyer, Claudine Niederst, Khalil Bourahla, Georges Noel

**Affiliations:** 1 Radiotherapy department, Centre de lutte contre le Cancer Paul Strauss, 3, rue de la Porte de l’Hôpital, Strasbourg Cedex, BP 42, 67065, France; 2 Department of Hematology and Oncology, Hôpital Civil, 1 place de l’Hôpital, Strasbourg, 67098, France; 3 Nuclear Medicine department, Centre de lutte contre le Cancer Paul Strauss, 3, rue de la Porte de l’Hôpital, Strasbourg Cedex, BP 42, 67065, France

**Keywords:** Hodgkin’s lymphoma, IMRT, Dose distribution

## Abstract

**Purpose:**

To analyze the outcome and dose distribution of intensity-modulated radiation therapy (IMRT) by helical tomotherapy in women treated for large supradiaphragmatic Hodgkin’s disease.

**Material and methods:**

A total of 13 patients received adjuvant radiation at a dose of 30 Gy to the initially involved sites with a boost of 6 Gy to those areas suspected of harboring residual disease on the simulation CT scan.

**Results:**

With a median follow-up of 23 months, the two-year progression-free survival was 91.6%, and the 2- and 3-year overall survivals were 100%. We did not report any heart or lung acute side effects. The conformity index of PTV (Planning Target Volume) was better for IMRT than for 3D-CRT (p=0.001). For the breasts, lungs, heart, thyroid and esophagus, the volume distributions favored the IMRT plans. For the breasts, the V_20Gy_, V_25Gy_ and V_30Gy_ were 1.5, 2.5 and 3.5 times lower, respectively, for IMRT than for 3D-CRT. For the lung tissues, the V_20Gy_ and V_30Gy_ were 2 times and 4.5 times lower, respectively, for IMRT than for 3D-CRT. For the heart, the V_20Gy_ and V_30Gy_ were 1.4 and 2 times lower, respectively, for IMRT than for 3D-CRT. For the esophagus, the V_35Gy_ was 1.7 lower for IMRT than for 3D-CRT, and for the thyroid, the V_30Gy_ was 1.2 times lower for IMRT.

**Conclusion:**

IMRT by helical tomotherapy improved the PTV coverage and dramatically decreased the dose in organs at risk. The treatment was well tolerated, but a longer follow-up is necessary to prove a translation of these dosimetric improvements in the outcome of the patients.

## Introduction

Early-stage Hodgkin lymphoma treated with a combination of chemotherapy and radiotherapy has an excellent clinical outcome, with overall survival reaching 90% [1-4]. However, late effects can dramatically affect the quality of life or be life-threatening in these survivors [5]. These late complications, including cardiovascular effects and secondary cancer, are due to large radiation doses and fields and also to chemotherapy, both of which lead to cardiovascular toxicity [6,7] and increase the risk of secondary cancer [6,8-11]. Depending on the follow-up, the incidence rate ranges from 4.6% to 20% [10,12-17]. The incidence of hematologic cancers is the most important, with standardized incidence ratios (SIRs) between 1.5 and 30 times higher than for solid tumors [10,11,18]. The occurrence of a secondary cancer is more frequent for women than men [10,19], mainly because of secondary breast cancers. Several risk factors have been associated with radiation-induced breast cancers, such as the age at treatment [20], higher irradiation dose [21,22] and the irradiation volumes [16,22].

Because radiotherapy remains a cornerstone in the treatment of Hodgkin lymphoma, some improvements have been developed recently to decrease the risk of side effects: i) decreasing the delivered dose, ii) irradiating the initially involved fields, and iii) using modern radiation techniques, such as intensity-modulated radiation therapy (IMRT) [2,3,23-26]. The decrease in the irradiated volume in the breasts could potentially decrease the risk of secondary breast cancer, thereby improving the prognosis in these patients [8]. We propose to demonstrate that using the helical tomotherapy HiArt system can achieve both the goal of improving the dose distribution to large or bulky PTV and that of sparing organs at risk compared with 3D radiation therapy (3D-RT) in young women who are particularly at risk for secondary breast cancer. We present the results of this study comparing dosimetric plans for 3D-conformal radiation therapy (3D-CRT) and for IMRT by helical tomotherapy (IMRT-HT) in women with Hodgkin disease.

## Materials and methods

### Patients and methods

A total of 13 patients with a median age of 29.7 years (17–53 years) at diagnosis were treated for newly diagnosed supradiaphragmatic stage II Hodgkin’s disease. Twelve patients had stage IIA, and one had stage IIB. All patients had cervical and mediastinal lymph node involvement. The initially involved nodal areas were described according to Mountain and Dresler’s international classification [27] for mediastinal node areas and the Gregoire et al. classification for head and neck node involvement [28]. Internal mammary chain and axillary lymph nodes were involved in four patients (Table 1). All patients received chemotherapy containing adriamycin, bleomycin, vinblastine and dacarbazine (ABVD): 1 patient received 3 cycles, 9 patients received 4 cycles, and 2 patients received 6 cycles. One additional patient was treated with 2 cycles of ABVD followed by 2 cycles of BEACOPP (bleomycin, etoposide, adriamycin, cyclophosphamide, vincristine, procarbazine and prednisone).

**Table 1:** Involved lymph nodes areas (X) and those suspects of no sterilization after chemotherapy (Xo)

**Lymph node areas**		**Patient 1**	**Patient 2**	**Patient 3**	**Patient 4**	**Patient 5**	**Patient 6**	**Patient 7**	**Patient 8**	**Patient 9**	**Patient 10**	**Patient 11**	**Patient 12**	**Patient 13**
Cervical lymph node areas	2R					**X**			**X**		**Xo**			
	3R	**X**				**Xo**	**Xo**		**X**		**X**		**X**	
	4R	**X**	**Xo**			**Xo**	**Xo**	**X**	**Xo**		**Xo**	**X**	**Xo**	**X**
	5R	**X**				**X**			**Xo**		**X**			
	2 L	**Xo**									**X**			
	3 L	**Xo**			**X**		**X**			**X**	**Xo**			
	4 L	**Xo**		**Xo**	**Xo**		**Xo**	**Xo**	**X**	**X**	**Xo**	**Xo**	**Xo**	**Xo**
	5 L	**X**		**Xo**						**X**		**X**		
Mediastinal lymph node areas	1R	**Xo**	**Xo**	**Xo**		**Xo**	**X**			**X**		**X**	**X**	**X**
	2R	**Xo**	**Xo**	**Xo**	**Xo**	**Xo**	**X**			**X**	**X**	**X**	**X**	**X**
	1 L	**Xo**		**X**	**Xo**	**X**	**X**					**X**	**X**	**X**
	2 L	**Xo**		**X**		**X**	**X**		**X**		**X**	**X**	**X**	**X**
	3A	**Xo**	**Xo**	**X**		**Xo**	**X**	**X**	**X**	**X**		**X**		**X**
	3P									**X**		**X**		**X**
	4R		**Xo**				**X**			**X**	**X**	**Xo**	**X**	**X**
	4 L		**X**				**X**				**X**			**X**
	5	**X**		**Xo**	**Xo**		**X**	**X**	**Xo**	**Xo**	**X**	**Xo**	**X**	**Xo**
	6	**X**		**Xo**	**Xo**		**X**	**Xo**	**Xo**	**Xo**	**X**	**Xo**	**X**	**Xo**
	7									**X**	**X**			
	8													
	10R						**X**		**X**	**X**	**X**			
	10 L													
	Para cardiac R	**X**												
Axillary lymph node area	R						**X**							
	L			**X**			**X**			**Xo**				
Internal mammary chains	R						**X**					**Xo**		
	L						**X**							

### Simulation

A customized immobilization mask was used for all patients. The patients underwent two virtual simulations on a dedicated computed tomography (CT) instrument (General Electric™ Lightspeed QXI) using 3.75 mm slices. The first was performed before any chemotherapy with contrast enhancement, and the second, without injection, was performed 15 days after the completion of the chemotherapy and 15 days before the start of radiotherapy; the same position and mask (no masks were remade) were used. The CTs were performed using a free breath schedule; patients were placed in the supine position with both arms along the body. Furthermore, all the patients underwent an ^18^ F-FDG-PET scan with 3.27 mm slices before any treatment (General Electric Discovery ST); this scan was used for simulation and delineation. The position for the PET scan was equivalent to that used for the simulation CTs.

### Volume definition

The pre-chemotherapy CT and PET were fused with the pre-RT CT. Contouring was performed with Focal (Elekta AB, Stockholm, Sweden) for 3D-CRT and IMRT-HT. The clinical target volume (CTV) and planning target volume (PTV) were determined according to INRT guidelines [29,30]. The PTV was obtained by adding 1-cm isotropic margins to the clinical target volume [30]. The ^18^ F-FDG-PET scan was used to improve the detection of initially involved lymph nodes [31]. The organs at risk (OARs) were delineated, including the heart, spinal cord, thyroid, esophagus, lungs and breasts.

### Treatment planning and dosimetric parameters

The dosimetries were calculated using the Xio (Elekta AB, Stockholm, Sweden) and Tomotherapy planning systems (Tomotherapy Incorporated, Madison, WI, USA). The dosimetric comparison was performed with Artiview (Aquilab, Lille, France).

The radiotherapy delivered 30 Gy in 15 fractions of 2 Gy, five days a week to PTV_30Gy_, with boost of 6 Gy in three fractions of 2 Gy to PTV_36Gy_. The PTV was planned to receive at least 95% of the prescribed dose according to ICRU 50 and 62 [32,33]. For both the 3D-CRT and IMRT plans, the data were collected with respect to the median (D50%), near-max (D2%) and near-min (D98%) doses according to the ICRU 83 [34]. The conformity (CI), homogeneity (HI) and coverage (CO) indices of the plans were calculated [35]. The CI was defined by the ratio between the reference isodose volume (RIV) and the PTV, and the HI was defined by the ratio between D2% (near-max dose) received by the target volume and the reference dose. We also compared the coverage index (CO) corresponding to the ratio between D98% (near-min dose) received by the target volume and the reference dose. The V_5Gy_, V_20Gy_ and V_30Gy_ for both lungs minus the PTV were limited, respectively, to 60%, 30% and 20% [36-40]. The dose limits also included a maximal spinal cord dose limit < 45 Gy and a mean heart dose < 20 Gy. For the breast, the median, mean, maximal and minimal doses and the volumes receiving total doses of 1 to 36 Gy were recorded for each breast and for a volume summing both volumes.

The 3D-CRT field set-up was performed with two opposed parallel antero-posterior fields equally powered with 6 and 25 MV photon beams. For the IMRT, the field width, pitch and modulation factors for the treatment planning optimization were 2.5 to 5 cm, 0.287 and 2.5, respectively.

### Follow-up

The patients were seen in consultation every two months during the first year and every four months during the 2^nd^ and 3^rd^ years. CT and PET scans were performed at least every six months. A lung function test (LFT) and cardiac ultrasound with the calculation of the left ventricular ejection fraction (LVEF) were performed at least every year and were compared with the values obtained before irradiation.

### Statistical analysis

The dosimetric parameters of each patient were compared with the non-parametric equivalent of a paired t-test for matched observations (Wilcoxon test). The threshold for statistical significance was p < 0.05. All statistical analyses were performed using Statview 5.1 software (version 5.1 SAS Institute In.).

## Results

The median follow-up of the 13 patients was 23 months (range: 16–48). The median CTV and PTV were 272 mL (range: 155–950) and 970 mL (range: 512–2666), respectively. We observed two relapses at 15 and 28 months. The first relapse arose in a non-irradiated site (with no fixation of ^18^FDG and no adenopathy in the first exam of the site), and the patient was treated with a new irradiation. The patient was alive at 48 months. The second relapse appeared at the irradiated site and was treated by chemotherapy. The two-year progression-free survival was 91.6%, and the 2- and 3-year overall survivals were 100%. The median number of LCVE controls was 2 (1–4). No cardiac dysfunction was observed, with a mean LVEF of 63% (60–70) before irradiation and 63% (55–72) at the last control. The median number of LFT was 2 (1–4). No change of the LFT at 1 year was observed, and no patient complained of breath dysfunction.

### Dose distribution

#### Target dosimetry (Table 2)

**Table 2:** PTV dose-constraints and indexes for each patient

**Patient**	**V95%**		**D98%**		**D50%**		**D2%**		**CI**		**HI**		**CO**	
	**3D-CRT**	**IMRT HT**	**3D-CRT**	**IMRT HT**	**3D-CRT**	**IMRT HT**	**3D-CRT**	**IMRT HT**	**3D-CRT**	**IMRT HT**	**3D-CRT**	**IMRT HT**	**3D-CRT**	**IMRT HT**
*1*	95.6	96.2	91.3	93.05	102	99.9	106.6	104.1	2.3	1.2	1.1	1.1	0.6	0.8
*2*	96.8	94,9	92.9	90.7	100.7	99.9	104.7	102.8	2.4	1.1	1.1	1.1	0.7	0.8
*3*	92.3	98.1	82.5	95.2	100	100	104.4	103.05	2.3	1.3	1.1	1.1	0.5	0.7
*4*	94.3	99.4	92.3	97.8	99.4	100.1	107.3	102.1	2.8	1.3	1.1	1.1	0.8	0.9
*5*	91.9	97.5	90.4	94.4	99.1	99.8	105	102.3	2.3	1.3	1.1	1.1	0.6	0.7
*6*	76.8	94.6	86.3	90.6	97.7	101.2	104.7	105.8	2.6	1.3	1.1	1.1	0.4	0.7
*7*	94.5	95.3	88.1	92.3	103.05	100	106	101.6	2.8	1.1	1.2	1.1	0.6	0.8
*8*	92.2	94.9	87.5	91.1	101.6	100	108.6	102.9	2.4	1.1	1.1	1.1	0.5	0.7
*9*	96.1	94.3	93.3	90.5	99.6	99.7	103.7	102.9	2.4	1.1	1.1	1.1	0.6	0.8
*10*	94.1	96.8	90.8	93	99.8	99.7	104.5	102.5	2.3	1.3	1.1	1.1	0.7	0.7
*11*	97.8	94.1	94.6	90	101.7	99.8	104.9	103	3.1	1.1	1.1	1.1	0.5	0.7
*12*	97.2	95.1	93.7	91.6	102	100	106.6	103.4	3.0	1.1	1.1	1.1	0.8	0.8
*13*	97.7	94.5	94.5	90.5	101.4	99.8	105.4	102.9	3.0	1.2	1.1	1.1	0.8	0.8
*mean*	93.7	95.8	90.6	92.4	100.6	100	105.6	103	2.6	1.2	1.1	1.1	0.6	0.8
*median*	94.5	95.1	91.3	91.6	100.7	99.9	105	102.9	2.4	1.2	1.1	1.1	0.6	0.8
*(p)*	NS		NS		NS		0.002		0.001		NS		0.001	

The conformity index was better for IMRT than for 3D-CRT, at 1.2 and 2.4, respectively (p=0.001). The median cover index was 0.8 (range: 0.4-08) for IMRT and 0.6 (range 07–0.9) for 3D-CRT (p=0.001). The homogeneity indices were not significantly different between the two plans. The mean V95% values for IMRT and 3D-CRT were 95.8% (range: 94.1-99.4%) and 93.7% (range: 76.8-97.8%), respectively, with no significant difference between the two plans. The IMRT plans resulted in significantly lower D2% values compared with 3D-CRT, at 102.9% (range: 103.7-108.6%) and 105% (range: 101.6-105.8%), respectively (p=0.002). The D50% and D98% were not significantly different between IMRT and 3D-CRT.

#### Breasts (Table 3, Figures 1A, 2)

**Figure 1:**
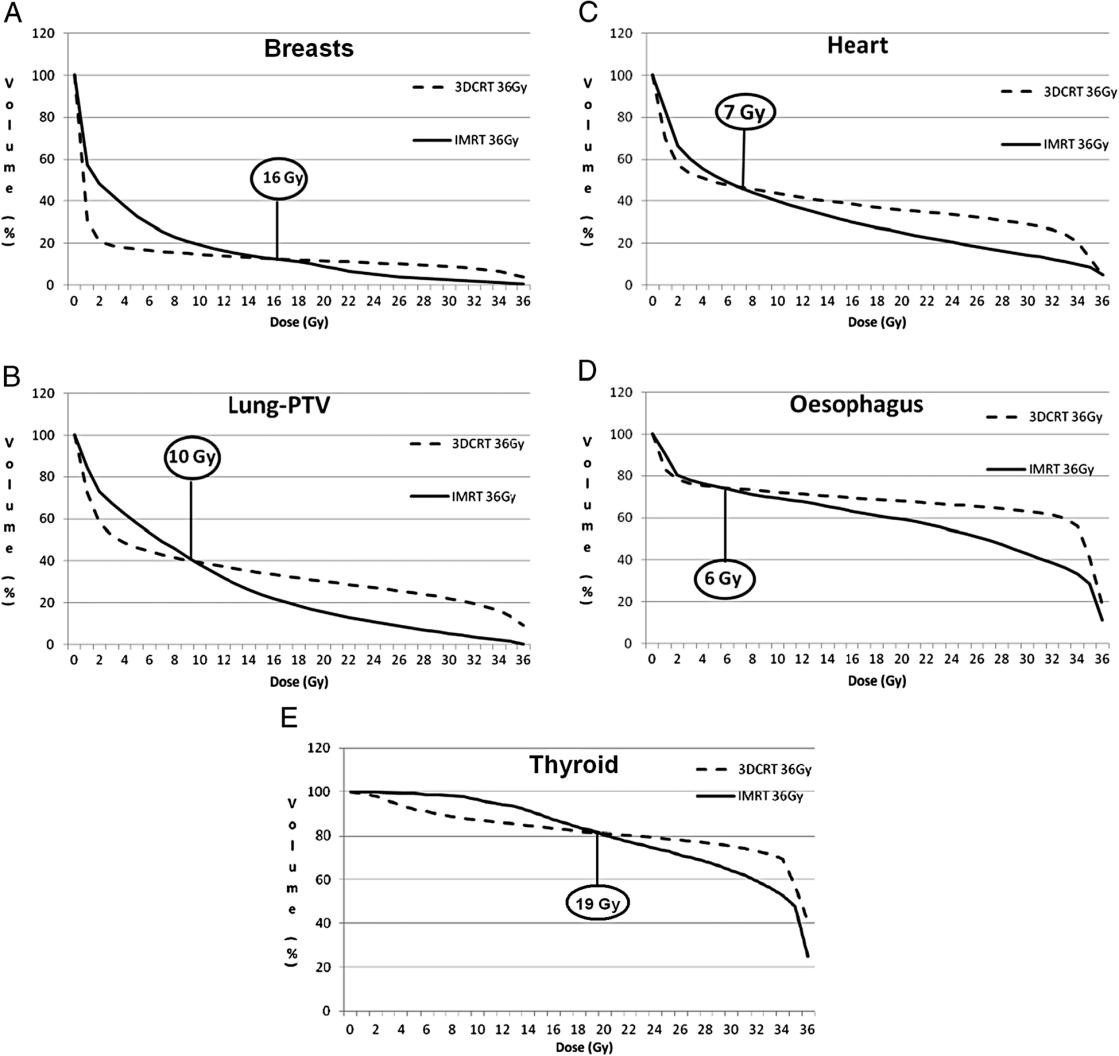
**Intersect of average DVHs of IMRT and 3D-CRT for organs at risk. A**: intersect of average DVHs for both breasts, **B**: intersect of average DVHs for lungs minus PTV; **C**: intersect of average DVHs for heart; **D**: intersect of average DVHs for oesophagus; **E**: intersect of average DVHs for thyroid.

**Figure 2:**
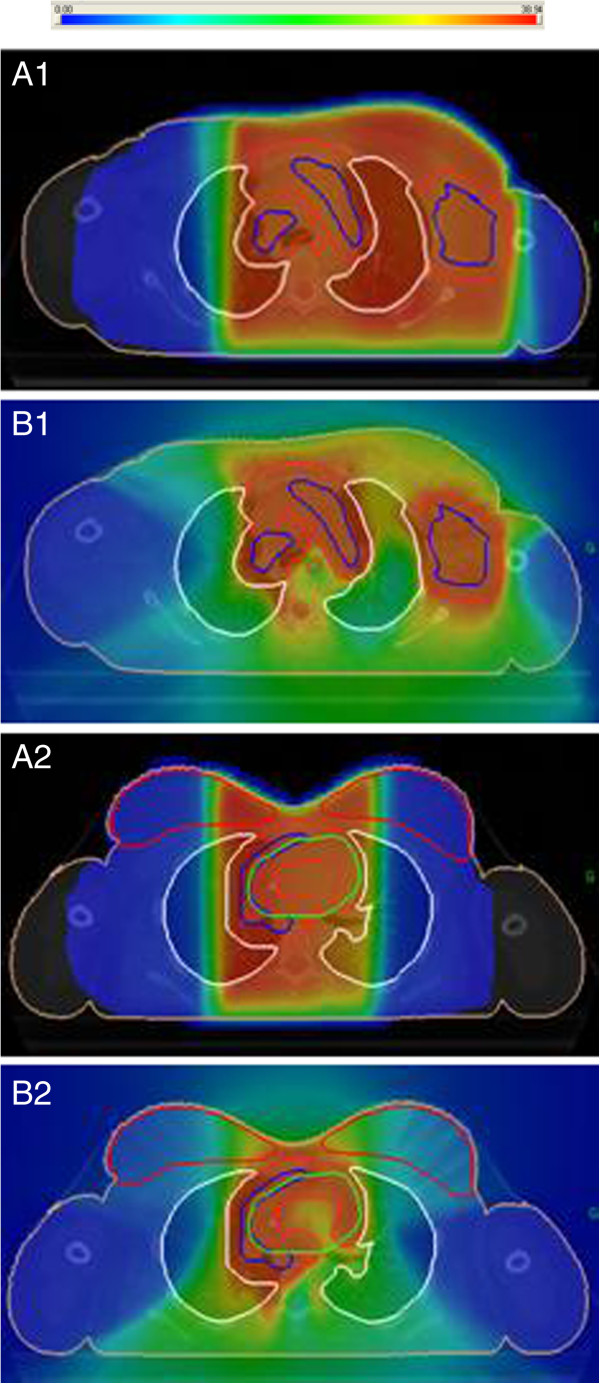
Dose distribution for the 3D-CRT (A1-A2) and IMRT techniques (B1-B2).

**Table 3:** Dose-comparisons into organs at risk according to radiation therapy technique

**Factors / Dose (Gy)**		**3D-CRT**		**IMRT**		**( *p *)**
		**Median**	**Mean**	**Median**	**Mean**	
**Breasts**	*Dmax*	38.6	38.3	35.3	33.6	0.001
	*Dmin*	0.001	0.02	0.3	0.8	0.001
	*Dmean*	2.7	4.9	4.7	5.7	0.05
	*V5 Gy*	9.9	17.2	27	32.7	0.001
	*V20 Gy*	5.7	11.6	3.9	9.1	0.002
	*V25 Gy*	5.05	10.4	1.2	4.6	0.001
	*V30 Gy*	4.05	8.9	0.2	2.6	0.001
**Right breast**	*Dmax*	37.7	34.6	32.7	30.3	0.01
	*Dmin*	0.001	0.02	0.3	0.8	0.001
	*Dmean*	2.1	3.8	4.4	5.2	0.007
	*V5 Gy*	7.8	13.1	25.5	29.7	0.001
	*V20 Gy*	4.3	8.5	2.6	7.8	NS
	*V25 Gy*	3.5	7.5	1.04	4.3	0.004
	*V30 Gy*	2.7	6.4	0.2	2.3	0.002
**Left breast**	*Dmax*	38.3	35.5	34	31.8	0.02
	*Dmin*	0.001	0.08	0.3	1.5	0.001
	*Dmean*	3.2	6	4.6	6.2	NS
	*V5 Gy*	11.1	21.3	31.8	35.8	0.007
	*V20 Gy*	6.4	14.4	3.6	10.1	0.002
	*V25 Gy*	5.5	13	1.5	4.9	0.002
	*V30 Gy*	4.4	11.1	0.4	2.8	0.002
**Lung-PTV**	*Dmax*	38.6	38.4	36.8	36.9	0.003
	*Dmin*	0.1	0.2	0.3	0.7	0.002
	*Dmean*	12	12.4	9.2	9.7	0.003
	*V5 Gy*	47.1	46.3	56.4	57.7	0.001
	*V20 Gy*	28.2	30.3	12.2	15.5	0.001
	*V30 Gy*	19.4	22.1	4.9	5.3	0.001
**Heart**	*Dmax*	37.3	37.1	38.1	38	0.01
	*Dmin*	0.3	0.6	0.7	1.3	0.001
	*Dmean*	13	13.9	11.2	11.5	0.01
	*V20 Gy*	32.3	35.7	20.7	25	0.002
	*V30 Gy*	23.1	29	11.1	14.4	0.001
**Oesophagus**	*Dmax*	37.3	37.3	36.9	36.9	NS
	*Dmin*	0.4	3.0	0.8	1.7	0.02
	*Dmean*	23.3	24.6	20.8	21.5	0.002
	*V35 Gy*	48.4	41	26.7	28.5	0.02
**Thyroid**	*Dmax*	37.2	37.2	36.9	37.1	NS
	*Dmin*	3.4	13.9	12.6	15.1	NS
	*Dmean*	34.6	30.1	31.6	29.4	NS
	*V30 Gy*	87.7	75.1	72.7	64.1	0.003
**Integral dose**	*Mean (Gy.L)*	-	179.0	-	176.8	NS

For both breast volumes, the median maximal dose was significantly lower for IMRT than for 3D-CRT, at 35.3 Gy and 38.6 Gy, respectively (p = 0.001). However, the median mean dose was significantly higher for IMRT: 4.7 Gy compared with 2.7 Gy for 3D-CRT (p = 0.05). As expected, the volumes receiving the highest dose were lower for IMRT; we observed a crossing of the mean DVH curves at 16 Gy (Figure 1). For both breasts, the volume distribution was significantly better for 3D-CRT from 1 to 11 Gy (p ≤ 0.05) and for IMRT from 19 to 36 Gy (p ≤ 0.04). Additionally, the median V_20Gy_, V_25Gy_ and V_30Gy_ values were significantly lower for IMRT compared with 3D-CRT at 3.9% and 5.7% (p = 0.002), 1.2% and 5% (p = 0.001), and 0.2% and 4% (p = 0.001), respectively. However, the V_5Gy_ was significantly higher for IMRT compared with 3D-CRT, at 27% and 9.9% (p = 0.001), respectively.

#### Lung (Table 3, Figure 1B)

The mean average and maximal doses were significantly higher for 3D-CRT than for IMRT, at 12.4 and 38.4 Gy and 9.7 and 36.9 Gy, respectively (p = 0.003 for both). The mean V_20Gy_ and V_30Gy_ were significantly higher for 3D-CRT than for IMRT, at 30.3 and 22.1% and 15.5 and 5.3%, respectively (p = 0.001 for both). However, the mean V_5Gy_ was significantly lower for 3D-CRT than for IMRT, at 46.3% and 57.7%, respectively (p = 0.001). The lung volumes receiving doses from 1 to 8 Gy were significantly lower for 3D-RT (p = 0.02). Between 9 and 11 Gy, the volume distributions were not significantly different. Between 12 and 36 Gy, the lung volumes were significantly lower for IMRT (p ≤ 0.01). The curves intersected at a dose of 10 Gy.

#### Heart (Table 3, Figure 1C)

The mean average dose was significantly higher for 3D-CRT than for IMRT, at 13.9 and 11.5 Gy, respectively (p = 0.01), although the maximal dose was significantly lower for 3D-CRT than for IMRT, at 37.1 Gy and 38 Gy, respectively (p = 0.01). The mean V_20Gy_ and V_30Gy_ were significantly higher for 3D-CRT than for IMRT, at 35.7 and 29% and 25 and 14.4%, respectively (p = 0.002 and p = 0.001, respectively). The heart volumes receiving doses of 1 or 2 Gy were significantly lower for 3D-RT (p < 0.009). Between 3 and 11 Gy and for 35 and 36 Gy, the mean volume distributions were not significantly different. Between 12 and 34 Gy, the mean volume distributions were significantly lower for IMRT (p ≤ 0.04). The curves intersected at a dose of 7 Gy.

#### Esophagus (Table 3, Figure 1D)

The mean average dose was significantly higher for 3D-CRT than for IMRT, at 24.6 and 21.5 Gy, respectively (p = 0.002). The mean maximal doses were not significantly different for 3D-CRT and for IMRT. The mean V_35Gy_ was significantly higher for 3D-CRT than for IMRT, at 41% and 28.5%, respectively (p = 0.02). Between 1 and 9 Gy and for 36 Gy, the mean volume distributions were not significantly different. Between 10 and 35 Gy, the mean volume distributions were significantly lower for IMRT (p ≤ 0.04). The curves intersected at a dose of 6 Gy.

#### Thyroid (Table 3, Figure 1E)

The mean average, maximum and minimal doses were not significantly different for 3D-CRT compared with IMRT, at 30.1 and 29.4 Gy, 37.2 and 37.1 Gy and 13.9 and 15.1 Gy, respectively. The mean V_30Gy_ was significantly higher for 3D-CRT than for IMRT, at 75.1 and 64.1%, respectively (p = 0.0033). For 1 and 2 Gy, between 18 and 20 Gy and for 36 Gy, the mean volume distributions were not significantly different. Between 3 and 17 Gy, the mean volume distributions were significantly lower for 3D-CRT (p ≤ 0.02). Between 21 and 35 Gy, the mean volume distributions were significantly lower for IMRT (p ≤ 0.02). The curves intersected at a dose of 19 Gy.

## Discussion

This study reports a dosimetric comparison of a series of 13 women with bulky early-stage Hodgkin lymphoma treated with INRT and IMRT using tomotherapy. This study is the third publication comparing IMRT and conformal 3D radiotherapy; a case report previously compared both techniques [41], and one series of 10 women comparing four irradiation plans was recently published [8]. In our series, the volumes were large (bulky tumors) and needed extensive radiation fields, leading to a difficult conformal treatment able to spare numerous organs at risk, such as the heart, lung, thyroid and spine. All plan comparisons were performed before the treatment, and overall the patients were treated with IMRT. Because of the excellent outcome of the patients with early-stage Hodgkin disease and the known risk of ionizing radiation, decreasing the treatment was performed. A decrease in the total dose was the first step [23]; the second step was the adaptation of the irradiated volume from the involved fields to the initial involved node areas [3,29,30,42-44]. A “Mantelet” field was progressively avoided. The last step to improve the dose distribution was the use of IMRT or proton therapy or of respiratory gating [24,25,30,45-49]. All these techniques aim to achieve better homogeneity of the target dose distribution, which we proved in our study as the conformity index was reduced by a factor of 2 between 3D-CRT and IMRT.

However, the treatment remains challenging. A recently published trial demonstrated that a combination of ABVD and radiotherapy improved disease-free survival compared with ABVD alone but that the overall survival was worse in the combination group [50]. The main reason for the increase in mortality with the combination treatment was the excess of causes of death other than Hodgkin disease in the combined treatment group [50]. The doses and radiation technique could be responsible for this loss of survival. However, in that study, the RT-fields used in the RT-arm were outdated, i.e., subtotal nodal irradiation, which is known to contribute to increase morbidity and mortality from cardio-toxicity and secondary cancers. Moreover, many of the deaths in the radiotherapy arm were clearly unrelated to radiotherapy [50]. A meta-analysis that pooled the results of trials comparing the combination of chemotherapy and radiotherapy and the same chemotherapy regimens alone showed that combined treatment was associated with a better disease-free survival and a higher survival rate. However, the chemotherapy schedules were not considered optimal [51].

The main causes of death after Hodgkin disease treatment include secondary cancers and lung and heart dysfunction. The cardiac pathologies induced by radiation are highly variable [52], and the delivered dose to the heart is a major factor of these complications [53,54]. However, the dose relationship that induces cardiac morbidity and cancer is a matter of debate as there is sufficient evidence to suggest a linear dose–response for cardiac mortality [55], with doses < 5 Gy most likely being less at risk than higher doses [56]. Decreasing the dose to the heart is a major goal, which can be reached with IMRT, even if the NTCP (normal tissue complication probability) is low at these doses [41].

For solid secondary neoplasms, breast and lung cancers are the most frequent types of malignant tumor [10,57]. The increase in the risk for breast cancer has been well described [58-64]. The roles of dose [64], volume [65] and age [10,16] are well known. The risk of secondary lung cancer is related to the combined treatment and, specifically for radiotherapy, the function of the dose and irradiated volume [66,67]. The dose–response relationships for secondary cancers also suggest a linear dose–response, with the exception of thyroid cancer [57,64].

The follow-up of the patients in this series could be considered to be short, but the risk of relapse is always quick; although we treated large tumors, we did not observe an increase in the relapse rate. Paumier et al. published comparable results with a longer follow-up [24,25]. Our series is the first study of women treated with tomotherapy IMRT, and we can suggest some conclusions. As expected, IMRT with tomotherapy decreased the median conformity index by twice, from 2.4 for 3D–CRT to 1.2 with IMRT, a highly significant difference. IMRT is feasible and well tolerated in terms of clinical tolerance and heart and pulmonary functions. Large doses were clearly demonstrated as responsible for the complications after Hodgkin disease. The dose distributions were clearly improved by IMRT. This improvement was obtained mainly for the higher doses that cause heart morbidity and induce cancer. For the breasts, doses greater than 20 Gy are at risk of inducing cancer. Van Leeuwen et al. showed a significantly higher breast cancer risk in patients receiving radiotherapy alone at a dose of ≥ 24 Gy [64]. Bhatia et al. also demonstrated that young patients given < 20 Gy to the breasts did not have a significantly higher risk of breast cancer compared with the controls [10]. The V_20Gy_, V_25Gy_ and V_30Gy_ were 1.5, 2.5 and 3.5 times lower, respectively, for IMRT than for 3D-CRT. For the lung tissues, the V_20Gy_ and V_30Gy_ were 2 times and 4.5 times lower, respectively, for IMRT than for 3D-CRT. For the heart, the V_20Gy_ and V_30Gy_ were 1.4 and 2 times lower, respectively, for IMRT than for 3D-CRT. For the esophagus, the V_35Gy_ was 1.7 lower for IMRT than for 3D-CRT, and for the thyroid, the V_30Gy_ was 1.2 lower for IMRT. Based on this list of classical constraints to critical organs, we demonstrated that IMRT can deliver a higher dose to the PTV and successfully decrease the highest dose in all the critical organs at risk of secondary cancer or dysfunction. The values of lung V_20Gy_ and heart V_30Gy_ were comparable with those previously published [8,24,41,48].

However, controlling the secondary appearance of breast cancer by ultrasound CT and magnetic resonance imaging beginning at least five to eight years after the completion of radiation therapy is recommended. For lung cancer, preventing smoking is recommended. Interestingly, the risk of mortality using the estimated 20-year survival for patients with solid secondary cancer was shown to be 72% compared with 80% for those who did not develop any secondary cancer, which was not significantly different [57]. Furthermore, a recently published study reported that conservative treatment followed by irradiation can be efficiently performed in patients with breast cancer after Hodgkin disease [68].

Some discussions have criticized the risk of secondary cancer with respect to the “bath” of low-to-moderate doses delivered to the patient’s body [49]. However, some arguments can reassure patients and physicians. The first complication in Hodgkin disease in cases similar to those in our series is cardiac side effects up to cardiac death. The decrease in the dose in this organ at risk is assumed to decrease the chance of this type of death or morbidity. The same conclusion can be reached for lung function complications. Some authors have translated the decrease in the dose in NTCP (normal tissue complication probabilities), showing the clear impact of the decrease in the dose in lowering complications [41,48].

Notably, the question of radiation-induced carcinogenesis remains controversial. In particular, the phenomenon of radiation hormesis at low radiation doses has attracted increasing attention [69]. Radiation hormesis is considered to be an adaptive response to the external stress of radiation exposure and is manifested in several cell lines in the form of reduced chromosomal aberrations and increased longevity.

Recently, Weber et al. showed by calculating the excess relative risk that decreasing the irradiation fields leads to a dramatic decrease in radiation-induced cancer. However, for comparable irradiation fields, the risks appeared higher for IMRT than for 3D-CRT. These calculations have been performed with linear and non-linear models, taking into account mainly the low dose volume risk [8]. However, two major clinical series for breast cancer demonstrated a dose-risk relationship [63,64]. Furthermore, clinical data have suggested that only a minority of tumors developed inside (<10%) or outside (11%) the PTV [70,71]. Rather, the majority of secondary cancers have been observed within the margin of the PTV [71] or at the field periphery [71,72]. This region of the dose or penumbra is rarely studied in the dose distribution and can highly vary according to the photon energy used for irradiation. This observation could suggest that radiation-induced cell death becomes dominant over carcinogenic mutations radiation dose increases. This hypothesis, thus, appears to contrast with the dose reduction developed recently and to not correlate with the clinical observation of Kirova et al., who showed that the dose levels at which secondary cancers are most likely to occur have not yet been clearly established. The authors showed that most reported cases of radiation-induced sarcomas after breast irradiation occurred at sites that had received doses of 60–80 Gy, with a minimal dose of 10 Gy [9].

Extrapolating the risk of radiation-induced carcinogenesis is an uncertain exercise. Data on radiation carcinogenesis are mainly derived from retrospective studies, with variable patient populations exposed to variable radiation doses with dosimetry that is often uncertain. In addition, a heightened risk of secondary malignancies may exist in these patients. In an extensive review of the literature, Suit et al. concluded that the experimentally observed heterogeneity in the induced secondary cancer risk indicated a large genetic role in the determination of risk in the individual [73]. Furthermore, due to the quite large and undefined heterogeneity in the patient populations studied, no precise quantification of the risk of radiation-induced secondary cancer is available at present [73]. Most of these series had difficulties in differentiating the pathological subtypes and dose distributions, which seems to be important data to take into account to ameliorate the predictive analysis. With respect to the risk of complications, a large series of children treated with ionizing radiation demonstrated that the risk of cancer induction was not clearly related to the dose. One third of those cancers arose in areas that received a low dose, one third in areas receiving a moderate doses, and one third in areas receiving a high dose [74]. In our series, specially conducted in women, we showed that IMRT was able to significantly avoid large tissue volumes receiving moderate to large doses at the cost of increasing the volume receiving a low dose. However, the integral dose is not increased by the IMRT technique with tomotherapy compared with 3D-CRT, as shown previously [41,75].

Another factor of confusion could have appeared because decreasing the dose to non-tumoral tissues will likely lead to a decrease in radiation-related non-cancerous disease. Thus, the absolute number of cancers could increase in the future by improving the survival of the global population.

A better understanding of the dose distributions and inducible secondary cancer for each organ is necessary to perform dosimetry with real dose constraints to protect against the development of secondary cancers. Additionally, a prudence principle is required. With respect to this goal, radiation oncologists are able to demonstrate some advantages of IMRT compared with 3D-RT.

## Conclusion

IMRT is an elegant treatment to irradiate large Hodgkin disease in women. It allows good local control to be achieved and has no acute side effects. Because of the decrease in the higher dose in most organs at risk, this therapy will hopefully decrease late complications. However, a longer follow-up is needed to definitively evaluate such an outcome.

## Consent

Written informed consent was obtained from the patient.

## Competing interests

The authors declare that they have no competing interests.

## Authors’ contributions

DA collected data, performed analysis and wrote article. GN design the study, analysed data and wrote article. PM, CN calculated dosimetries. All authors read and approved the final manuscript.
